# Genetic parameters of milk and lactation curve traits of dairy cattle from research farms in Thailand

**DOI:** 10.5713/ab.21.0559

**Published:** 2022-05-02

**Authors:** Santi Pangmao, Peter C. Thomson, Mehar S. Khatkar

**Affiliations:** 1 Sydney School of Veterinary Science, Faculty of Science, The University of Sydney, Camden, NSW 2570, Australia; 2 Dairy Cattle Research and Development Center, Pak Chong, Nakhon Ratchasima 30130, Thailand

**Keywords:** Dairy Cattle, Lactation Curve, Wood Model

## Abstract

**Objective:**

This study was aimed to estimate the genetic parameters, including genetic and phenotypic correlations, of milk yield, lactation curve traits and milk composition of Thai dairy cattle from three government research farms.

**Methods:**

The data of 25,789 test-day milk yield and milk composition records of 1,468 cattle from lactation 1 to 3 of Holstein Friesian (HF) and crossbred HF dairy cattle calved between 1990 and 2015 from three government research farms in Thailand were analysed. 305-day milk yield was estimated by the Wood model and a test interval method. The Wood model was used for estimating cumulative 305-day milk yield, peak milk yield, days to peak milk yield and persistency. Genetic parameters were estimated using linear mixed models with herd, breed group, year and season of calving as fixed effects, and animals linked to a pedigree as random effects, together with a residual error. Univariate models were used to estimate variance components, heritability, estimated breeding values (EBVs) and repeatability of each trait, while pairwise bivariate models were used to estimate covariance components and correlations between traits in the same lactation and in the same trait across lactations.

**Results:**

The heritability of 305-day milk yield, peak milk yield and protein percentage have moderate to high estimates ranging from 0.19 to 0.45 while days to peak milk yield, persistency and fat percentage have low heritability ranging from 0.08 to 0.14 in lactation 1 cows. Further, heritability of most traits considered was higher in lactation 1 compared with lactations 2 and 3. For cows in lactation 1, high genetic correlations were found between 305-day milk yield and peak milk yield (0.86±0.07) and days to peak milk yield and persistency (0.99±0.02) while estimates of genetic correlations between the remaining traits were imprecise due to the high standard errors. The genetic correlations within the traits across lactation were high. There was no consistent trend of EBVs for most traits in the first lactation over the study period.

**Conclusion:**

Both the Wood model and test interval method can be used for milk yield estimates in these herds. However, the Wood model has advantages over the test interval method as it can be fitted using fewer test-day records and the estimated model parameters can be used to derive estimates of other lactation curve parameters. Milk yield, peak milk yield and protein percentage can be improved by a selection and mating program while days to peak milk yield, persistency and fat percentage can be improved by including into a selection index.

## INTRODUCTION

Milk production in Thailand in 2018 was 1.25 million tons [1], most of which is used for consumption within the country. However, the current milk production is not enough to meet the domestic demand. Hence, milk production is the main focus in the dairy cattle breeding improvement programs in Thailand. Crossbreeding local Zebu cattle with exotic *Bos taurus* cattle, mainly Holstein Friesian (HF), is the main approach used to improve milk production in the country. Artificial insemination with HF semen for upgrading has been the main method of herd improvement. The dairy system is generally driven by small-scale farmers and cooperative organizations which have support from the Thai government [2]. In developing countries where there are often less data being recorded, genetic evaluation using the computationally simpler 305-day lactation yield record in an animal model is still commonly used.

Many methods have been used for calculating 305-day milk yield. The test interval method (TIM) is one of the standard methods approved by the International Committee for Animal Recording [3]. Milk production estimation by TIM is calculated as the area under the lactation curve up to a specific day, e.g. day 305, using a simple trapezoidal numerical integration method. A typical lactation curve involves a rapid increase after calving until it reaches the peak early in the lactation, followed by a decline in milk yield until the end of milk production. The lactation curve can be an important management tool as a summary of milk production in dairy cattle. Understanding the lactation curve and factors that influence its shape can help farmers to manage their herd effectively in terms of feed allocation, reproductive and health management which can be optimised to increase the milk yield. A number of mathematical models have been used to describe lactation curves. The Wood model [4] is one of the models that has been widely used due to the relatively few parameters to be estimated (three) and variety of model shapes [5]. It is more robust compared to other models when fitting lactation curve to irregular and infrequent test-day sampling regimes [6]. The inclusion of suitable and accurate estimates of genetic parameters of lactation curve characteristics into a breeding improvement program can be used to improve milk production.

This study used data from three dairy cattle research centres managed under the supervision of the Department of Livestock Development of Thailand. The aim of these centres is to study various aspects in dairy cattle raising in Thailand such as nutrition, farm management and breed improvement in order to gain and extend knowledge to smallholder farmers. The objective of this study was to estimate the genetic parameters, including heritabilities, genetic and phenotypic correlations, genetic trends of estimated breeding values (EBVs) of milk yield, lactation curve traits and milk composition of Thai dairy cattle in these three government research farms.

## MATERIALS AND METHODS

### Geography and climate conditions

**Figure 1:**
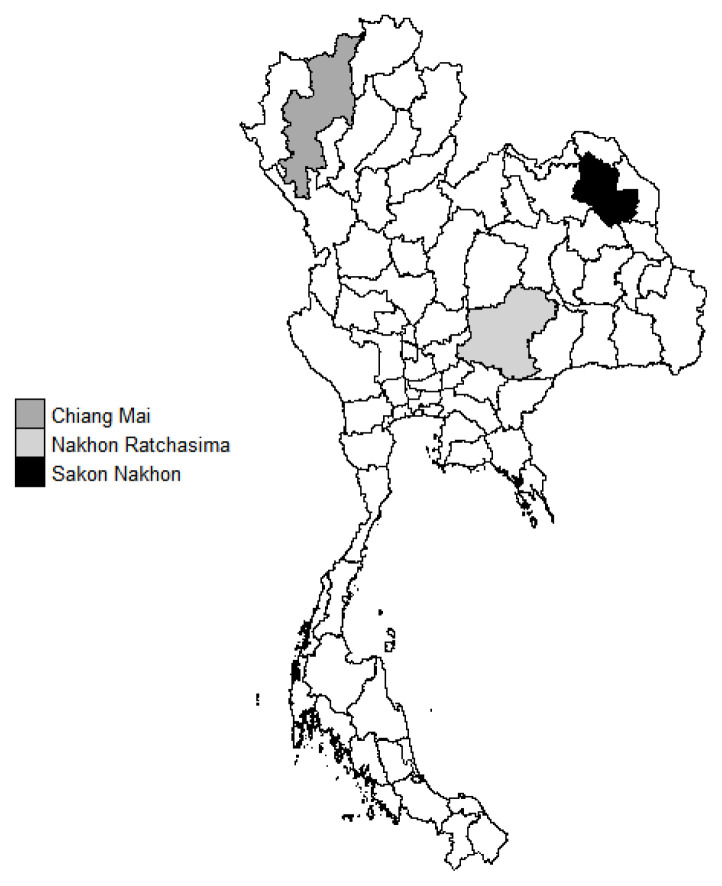
The geographic location (province) of three government dairy cattle research farms, included in this study, are highlighted in three different black and white scales on the map of Thailand.

Thailand is located in the tropics between latitudes 5°37′ N to 20°27′ N and longitudes 97°22′ E to 105°37′ E. The total area is about 513,000 km^2^. The weather conditions in Thailand are divided into three seasons, namely winter (mid-October to mid-February), summer (mid-February to mid-May) and rainy season (mid-May to mid-October) with an average temperature and relative humidity of 26°C and 74%, 29°C and 71%, and 28°C and 76%, respectively. The annual rainfall in most areas of the country is from 1,200 mm to 1,600 mm [7]. The three Thai government dairy cattle research farms where the data were obtained from were located in two main regions of Thailand (north and north-eastern). One farm in the north is located in Chiang Mai province (latitude 18°35′41.9″ N and longitude 98°51′53.1″ E) and the other two farms in the north-east are located in Nakhon Ratchasima (latitude 14°40′09.9″ N and longitude 101°26′46.7″ E) and Sakon Nakhon province (latitude 17°09′52.9″ N and longitude 104°02′07.8″ E) as shown in Figure 1.

### Description of data and herd management

The data from the three Thai government dairy cattle farms mentioned above were provided by the Bureau of Animal Husbandry and Genetic Improvement (BAHGI), Department of Livestock Development (DLD) in Thailand. The two farms located in Chiang Mai province and Nakhon Ratchasima province raised both Holstein-Friesian (HF) and crossbred HF while the third farm located in Sakon Nakhon province raised only crossbred HF. Upgrading local or Zebu breed with HF semen or natural mating with HF bulls has been used to improve productivity and maintain tropical insect and disease tolerance in these herds.

All animals in the three farms were raised under the same guidelines given by BAHGI. Nevertheless, some aspects were different such as feeding and health management because of the differences of locations, weather conditions, feed resources, farm machinery and disease prevalence. Fresh ruzi (*Brachiaria ruziziensis*) and Napier grass (*Pennisetum purpureum*) were fed during the rainy season while in the dry season, ruzi silage and hay were fed. In addition, fresh corn (*Zea mays*) and corn silage were used in Chiang Mai farm and Nakhon Ratchasima farm in some particular years (nutrient component of roughage and concentrate feed using in dairy cattle farm is described in Pangmao et al [2].

### Data and statistical analysis

The data comprised of 25,789 test-day milk yield and milk composition records of 1,468 cattle from Lactation 1 to 3 of HF and crossbred HF dairy cattle that calved between 1990 and 2015. The total number of sires, dams and individual animals in the pedigree file were 287, 1,237 and 4,753 respectively. The data consist of cow number, birth date, calving date, drying date, parity, lactation length, monthly or test-day records of milk yield, fat and protein percentage (FP and PP). The records of cows with less than three test-day milk yield records were excluded. The animals were classified into five breed groups (BG) based on the percentage of HF blood (HFB) as 1 (HFB≤75), 2 (75<HFB≤87.5), 3 (87.5<HFB≤93.75), 4 (93.75<HFB≤99.99) and 5 (HFB = 100). Calving months were grouped into three seasons, namely winter (November to February), summer (March to June) and rainy (July to October).

The Wood model [4] specifies the expected milk yield on day t of lactation, and has the following form, the shape depending on the three parameters:


$$W(t;k,b,c)=at^{b}e^{-ct}=\text{exp}(k+b{\text{log}}_{e}t-ct)$$

where *W*(*t*) is the theoretical or expected milk yield at day *t*, *k* is a scalar factor and equal to log_e_*a*, *b* is related to the rate of increase prior to the peak and c is related to the rate of decrease after the peak. The expected milk yield for cow-lactation *i* on days-in-milk *t* can be fitted as the following nonlinear mixed model:


$$y_{ij}=W(t_{ij};k_{i},b_{i},c_{i})+{ɛ}_{ij}$$

where *ɛ_ij_* is the random effect associated with records. Random effects for each cow’s lactation (*i*) are indicated as deviations (*K_i_*, *B_i_*, *C_i_*) from the fixed effect means:


$$k_{i}=k+K_{i},\;b_{i}=b+B_{i},c_{i}=c+C_{i}$$

and are assumed to have a multivariate normal distribution as follows:


$$\left(\begin{array}{c} K_{i}\\ B_{i}\\ C_{i} \end{array}\right)\sim N\;\left(\left(\begin{array}{c} 0\\ 0\\ 0 \end{array}\right),\left(\begin{array}{ccc} \sigma_{K}^{2} & \sigma_{KB} & \sigma_{KC}\\ & \sigma_{B}^{2} & \sigma_{BC}\\ & & \sigma_{C}^{2} \end{array}\right)\right),{ɛ}_{ij}\sim N(0,\sigma_{ɛ}^{2})$$

The parameters (*k*, *b*, *c*) were chosen over (*a*, *b*, *c*), as similar to the analyses by Hall [8], based on the preliminary fixed effects model, the former showed a better approximation to a multivariate normal distribution than did the latter (data not shown), a requirement for the nonlinear mixed model specification.

Cumulative milk yield to day *T* (day 305) was then obtained as $MILKW=\int_{0}^{T}W(t)dt$. Fitting of the Wood model as a nonlinear mixed model was conducted using the nlme package in R [9] and calculation of cumulative milk yield through use of the pgamma function in R. The parameters derived from Wood model were used to calculate lactation curve characteristics namely peak milk yield (PMILK = *e^k^*(*b*/*c*)*^b^e*^−^*^b^*), days to peak milk yield (DPMILK = *b*/*c*) and persistency (S = *c*^−(^*^b^*^+1)^) [4] (PSMILK = log*_e_*(S) = log*_e_*(*c*^−(^*^b^*^+1)^) = −(*b*+1)log*_e_c*.

The 305-day milk yield (MILKTI) calculated by using the TIM [10] with adjustment for the last term as follows:


$$\text{MILKTI}=I_{0}M_{1}+I_{1}\times \left(\frac{M_{1}+M_{2}}{2}\right)+I_{2}\times \left(\frac{M_{2}+M_{3}}{2}\right)+\ldots +I_{n-1}\times \left(\frac{M_{n-1}+M_{n}}{2}\right)+\frac{I_{n}M_{n}}{2}$$

where *M_1_*, *M_2_*, …, *M_n_* are the weights (kg), given to one decimal place, of the milk yield in the 24 hours of the recording day, *I*_1_, *I*_2_, …, *I_n_*_−1_ are the intervals, in days, between recording dates, *I*_0_ is the interval, in days, between the lactation period start date and the first recording date and *I_n_* is the interval, in days, between the last recording date and the end of the lactation period. The FP and PP were averaged over the lactation and used for subsequent analysis.

To obtain estimates of genetic parameters, univariate linear mixed models were fitted to each of the seven traits (MILKW, MILKTI, PMILK, DPMILK, PSMILK, FP, and PP), with a separate model for each of the three lactations. The fixed effects of each model comprised herd (Herd), BG, year and season of calving (YSOC); and the random effects were animal linked to a pedigree, and residual error. The form of each model was


$$y_{ijkl}=\mu +{\text{Herd}}_{i}+{\text{YSOC}}_{j}+{\text{BG}}_{k}+{\text{Anim}}_{i}+e_{ijkl}$$

where *y_ijkl_* is an observation of the trait on animal *l*; μ is the overall mean; Herd*_i_* is the fixed effect of herd (level, 1 to 3); YSOC*_j_* is the fixed effect of year and season of calving (level, 1 to 78); BG*_k_* is the fixed effect of BG (level, 1–5); Animl is the random animal effect; and *e_ijkl_* is the random residual effect. Heritability was estimated using REML estimates for $h^{2}=\sigma_{A}^{2}/(\sigma_{A}^{2}+\sigma_{e}^{2})$, where $\sigma_{A}^{2}$ and $\sigma_{e}^{2}$ are the additive genetic and residual variances. Estimated breeding values for each trait were also produced from the fitted univariate models.

Following this a repeatability model was fitted to all three lactations simultaneously, via the following model:


$$Y_{ijkl}=\mu +{\text{Herd}}_{i}+{\text{YSOC}}_{j}+{\text{BG}}_{k}+{\text{Lact}}_{m}+{\text{Anim}}_{l}+{\text{Pe}}_{l}+e_{ijklm}$$

where all terms are as defined previously, with the addition of Lact*_m_*, the fixed effect of lactation *m* (*m* = 1, 2, 3); and Pe*_l_*, the random ‘permanent environment’ term for animal *l*. For this model, heritability was estimated as $h^{2}=\sigma_{A}^{2}/(\sigma_{A}^{2}+\sigma_{pe}^{2}+\sigma_{e}^{2})$ where $\sigma_{pe}^{2}$ is the permanent environment variance, and repeatability as $r=(\sigma_{A}^{2}+\sigma_{pe}^{2})/(\sigma_{A}^{2}+\sigma_{pe}^{2}+\sigma_{e}^{2})$.

The set of univariable models at each lactation was ex tended to a series of pairwise bivariate models, for each pair of traits using the same terms as the univariate model, to allow estimation of genetic and phenotypic correlations between traits in the same lactation and in the same trait between two different lactations. Model fitting of these univariate and bivariate models was conducted using ASReml-R [11].

## RESULTS

The descriptive summary of milk yield, lactation characteristic traits and milk composition of lactations 1 to 3 is shown in Table 1. Milk yield from both models (MILKW and MILKTI) was highest in lactation 2 (3,685 and 3,682 kg) while lowest in lactation 1 (3,348 and 3,379 kg). Peak milk yield (PMILK) was lowest for lactation 1 at 14.95 kg while for lactation 2 and 3 were 17.52 kg and 17.49 kg, respectively. The cows in lactation 1 took longer to attain peak yield (DPMILK) than the cows in lactation 2 or 3 (49 days vs 39 days and 41 days), however, the persistency (PSMILK) of lactation 1 cows was higher than cattle in lactation 2 and 3 (6.68 vs 6.43 and 6.43). For milk composition, fat percentage (FP) of the cows in lactation 1 was less than lactations 2 and 3 (3.55% vs 3.64% and 3.64%) while protein percentage (PP) was the same for all lactation (3.04%).

### Heritability

The estimated additive genetic variance, residual variance and heritability for milk yield, lactation characteristic traits and milk composition of lactations 1 to 3 are shown in Table 2. In general, the heritability estimates of all traits from first parity cows were higher than in parity 2 and 3 except for FP that were lower (0.08, 0.25, and 0.07, respectively). This was due to a combination of greater genetic variances and smaller residual variances of traits in the first lactation. The heritability estimates of MILKTI in the first and third parity were similar (0.19). The heritability estimates of MILKTI and MILKW were similar (0.21 and 0.19) in lactation 1 while the heritability of MILKW was lower compared to MILKTI in lactation 2 and 3 (0.01 vs 0.12 and 0.08 vs 0.19, respectively). Lactation curve trait heritability estimates from cows in lactation 1 were higher than in lactation 2 and 3 whereas heritability of DPMILK and PSMILK were very low in lactation 2 and were zero or no detectable additive genetic variability in lactation 3. The heritability estimates of FP in lactation 1 and 3 was lower (0.08 and 0.07) than in lactation 2 although heritability of PP in lactation 1 was higher (0.45) compared to lactation 2 and 3 (both 0.22). The overall heritability using the repeatability model across three lactations was low to medium, ranging from 0.14 to 0.31 for all the traits while very low for DPMILK and PSMILK (0.03 and 0.04, respectively) as shown in Table 3. The overall repeatability for lactations 1 to 3 was low for most of the traits at between 0.22 and 0.34 (Table 3). MILKTI has the highest repeatability (0.45) while DPMILK and PSMILK has lowest repeatability (0.03 and 0.04, respectively).

### Genetic and phenotypic correlations among traits in lactation 1

The genetic correlation estimates between milk yield and lactation curve traits were moderate to high (0.57 to 0.99) except for the genetic correlation estimates between PMILK and DPMILK, and between PMILK and PSMILK (0.32 and 0.33) as shown in Table 4. The genetic correlations of milk composition with other traits were low and negative. PP has negative genetic correlations with other traits (−0.46 to −0.06) except for the genetic correlation with FP (0.34). The phenotypic correlations between MILKW, MILKTI and PMILK were in the high range from 0.70 to 0.92 while between DPMILK and PSMILK with other traits were negative and low (−0.07 to 0.25) except between DPMILK and PSMILK (0.93). The phenotypic correlations of FP and PP with other traits were negative and low (−0.11 to 0.11) except between FP and PP which was moderate (0.29).

### Genetic and phenotypic correlations among traits in lactation 2

The genetic correlation estimates between milk yield and lactation curve traits were high to very high (0.64 to 0.99) except for the genetic correlations between DPMILK and PSMILK with MILKTI (0.21 and 0.32 respectively) and between PSMILK and PMILK (0.23) as shown in Table 5. The genetic correlation estimates between FP and other traits were low to moderate (0.14 to 0.29) while between PP and other traits were negative (−0.46 to −0.06) except between PP and FP (0.34). The phenotypic correlation between MILKW with MILKTI, PMILK, PSMILK and PP were high ranging from 0.71 to 0.93 except between MILKW with DPMILK and FP were low (0.16 and 0.09, respectively) while the phenotypic correlation between MILKTI with other lactation curve characteristic traits were negative to moderate (−0.06 to 0.28). The phenotypic correlation between FP and PP with other traits were negative to low (−0.10 to 0.09) except for the phenotypic correlation between PP with FP and MILKW (0.28 and 0.93, respectively).

### Genetic and phenotypic correlations among traits in lactation 3

Table 6 shows the estimated genetic and phenotypic correlations of milk yield, lactation curve traits and milk composition of the cows in lactation 3. The genetic correlation between MILKW and MILKTI is moderate (0.45). The genetic correlation of PMILK, DPMILK, and PSMILK with MILKW and MILKTI are high (0.68 to 0.99) but moderate between MILKW and DPMILK (0.46) and highly negative genetic correlation between MILKW and PSMILK (−0.99). Most of the genetic correlation estimates of FP with other traits are moderate to high (0.44 to 0.99) except with DPMILK (−0.09) while mostly genetic correlations of PP with other traits are negative (−0.82 to −0.14) but with PMILK is 0.25. The phenotypic correlation between MILKW and MILKTI is 0.71. The phenotypic correlation estimates between MILKW and MILKTI with DPMILK and PSMILK are low to moderate ranging from 0.02 to 0.28 but between MILKW and MILKTI with PMILK are high (0.70 and 0.78). Phenotypic correlations between PMILK, DPMILK and PSMILK are negative low (−0.06 and −0.10) but between DPMILK and PSMILK is high (0.93). Phenotypic correlations of FP and PP with other traits are low (−0.04 to 0.08) except between FP and PP (0.25).

### Genetic and phenotypic correlation of each trait across lactations

The genetic correlation estimates of milk yield, lactation characteristics and milk composition between lactations 1 to 3 are shown in Table 7. Most of the genetic correlation estimates of the traits are high (0.75 to 0.99). However, the genetic correlations of DPMILK, PSMILK, and FP could not be estimated between lactations 1 and 3 and lactations 2 and 3. The phenotypic correlations for milk yield, lactation characteristics and milk composition of Thai dairy cattle between lactations 1, 2, and 3 are shown in Table 8. The phenotypic correlation of all traits is low for most of the traits.

### Time trends of trait estimated breeding values

**Figure 2:**
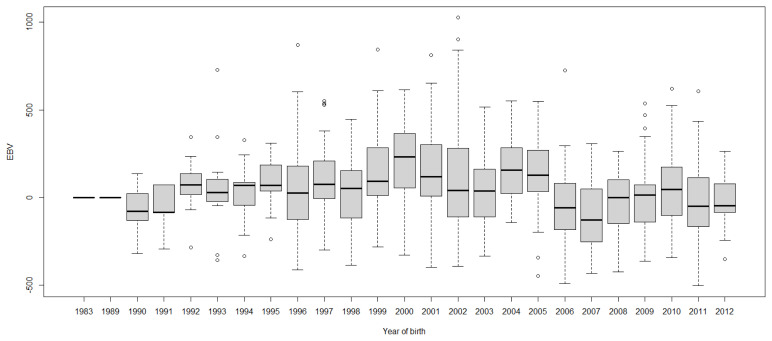
Boxplots of estimated breeding value (EBVs) by year of birth of cows for the Wood model cumulative 305-day milk yield in the first lactation. The genetic trend of the Wood model cumulative 305-day milk yield EBV shows an inconsistent pattern over the year of birth. From 1982 to 2000, the trend gradually increased and after that, the trend decreased with fluctuation.

**Figure 3:**
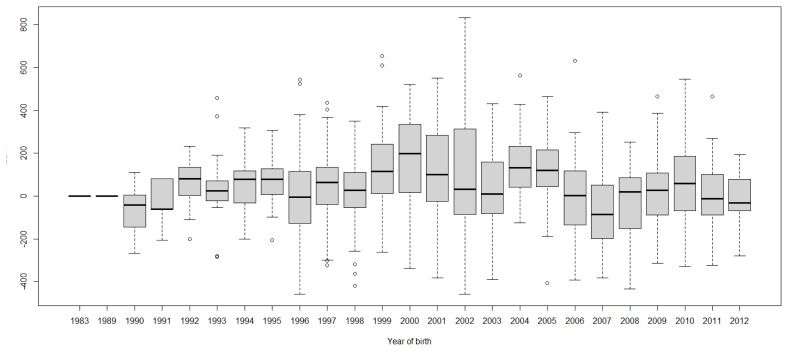
Boxplots of estimated breeding value (EBVs) by year of birth of cows for the test interval cumulative 305-day milk yield in the first lactation. The genetic trend of the test interval cumulative 305-day milk yield EBV shows an inconsistent pattern over the year of birth. From 1982 to 2000, the trend gradually increased and after that, the trend decreased with fluctuation.

**Figure 4:**
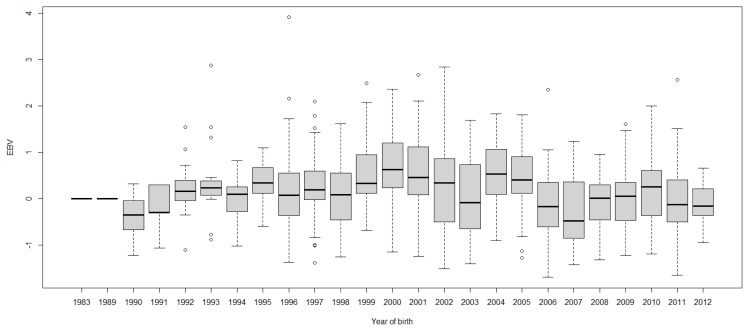
Boxplots of estimated breeding value (EBVs) by year of birth of cows for peak milk yield in the first lactation. The genetic trend of peak milk yield EBV shows an inconsistent pattern over the year of birth. From 1982 to 2000, the trend gradually increased and after that, the trend decreased with fluctuation.

**Figure 5:**
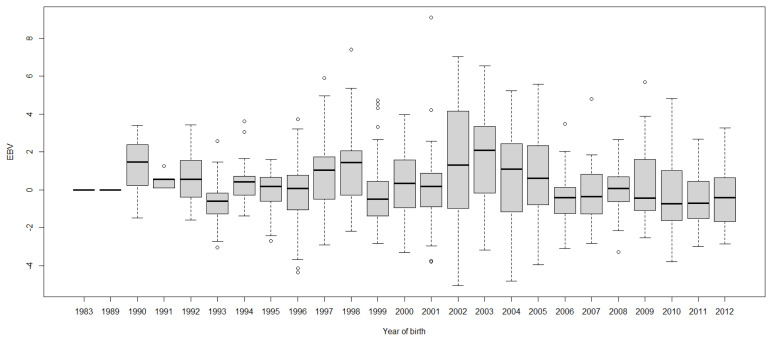
Boxplots of estimated breeding value (EBVs) by year of birth of cows for days to peak milk yield in the first lactation. The genetic trend of days to peak milk yield EBV shows an inconsistent pattern over the year of birth and from year 2003, the trend decreased with fluctuation.

**Figure 6:**
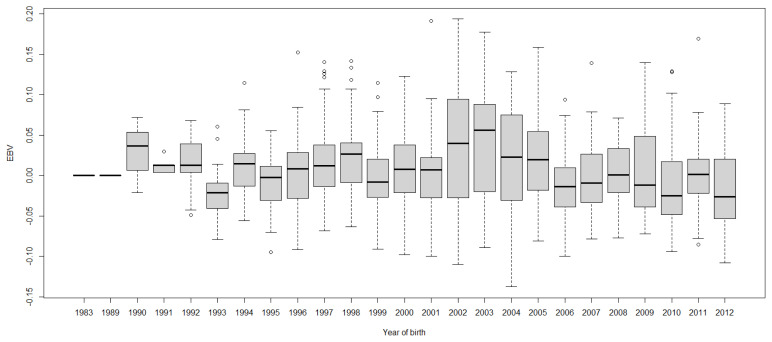
Boxplots of estimated breeding value (EBVs) by year of birth of cows for persistency in the first lactation. The genetic trend of persistency EBV shows an inconsistent pattern over the year of birth and from year 2003, the trend decreased with fluctuation.

**Figure 7:**
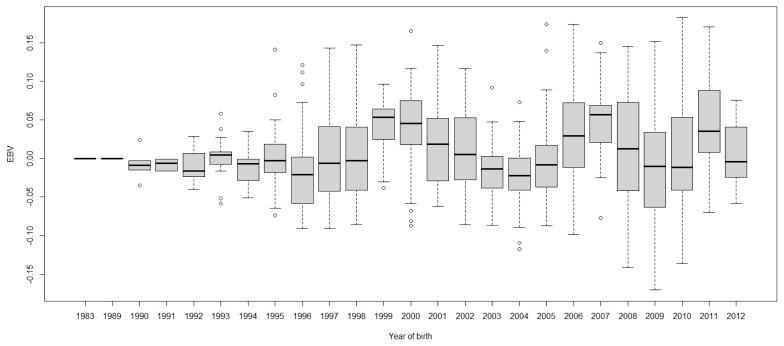
Boxplots of estimated breeding value (EBVs) by year of birth of cows for fat percentage in the first lactation. The genetic trend of fat percentage EBV shows an inconsistent pattern over the year of birth.

**Figure 8:**
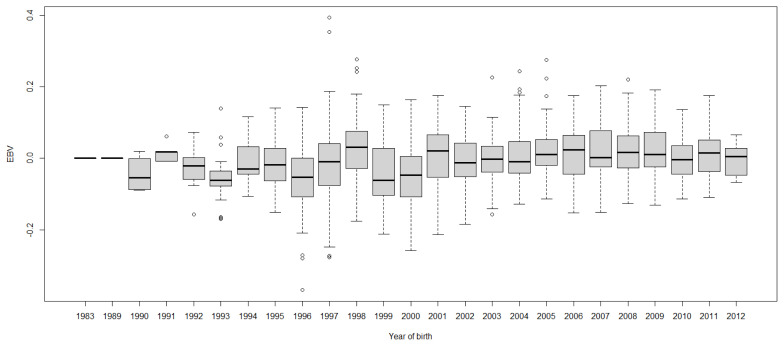
Boxplots of estimated breeding value (EBVs) by year of birth of cows for protein percentage in the first lactation. The genetic trend of protein percentage EBV shows an inconsistent pattern over the year of birth and from year 2000, the trend gradually increased with fluctuation.

Boxplots of cow EBVs over year of birth of all traits in lactation 1 are shown in Figures 2 to 8. None of the traits showed a systematic improvement over this period and did not show consistent trends. The EBV trend for most traits improved a little from 1982 to 2000, after that the EBV trend fluctuated and decreased during the later years. Boxplots of sire EBVs over the year of birth of all traits in lactation 1 are in the Supplementary document as Figures S1–S7. The sire EBVs of all traits showed the same inconsistent trend over years as the cow, however, there was an improvement in the trend in the last 10 years compared to 10 years before for most traits. The average sire EBV of MILKW in the 10 years before 2002 was −42.7 kg compared to +100.8 kg in the last 10 years after 2002.

## DISCUSSION

This study reported the genetic parameters of milk yield, lactation curve traits and milk composition from three government dairy farms estimated using data from the first three lactations of cows. For first lactation cows, milk yield and peak milk yield have moderate heritability, protein percentage has high heritability which can be improved by a selection scheme while days to peak milk yield, persistency and fat percentage have low heritability. The similarity of estimated milk yield and high genetic correlation between both methods suggested that either method can be used for a genetic improvement program in these herds. However, the Wood model has advantages over the test-interval method by requiring fewer test-day records, particularly if strategically selected [12]. In addition, lactation curve traits such as persistency can be estimated easily by the Wood model. However, even with the Wood model the estimates of heritability of milk yield and lactation curve traits in lactations 2 and 3 had high standard errors compared to the heritability estimates. Further study including more data from lactations 2 and 3 would be required to obtain more precise estimates. The positive genetic correlations between cumulative 305-day milk yield and peak milk yield, and between days to peak milk yield and persistency within a lactation implies that selection to improve peak milk yield would also improve milk yield, and that selection for improved days to peak milk yield would improve persistency as well. In addition, the high genetic correlations of all traits between lactations suggested that selection of favourable animals in the first lactation would also improve the corresponding trait in second and third lactations, and with these correlations being so high, may indicate they are essentially the same trait.

Both cumulative 305-day milk yields (MILKW and MILKTI) were similar within all the three lactations. The lowest mean MILKW was for lactation 1 compared with lactations 2 and 3 (3,348 kg vs 3,685 kg and 3,564 kg, respectively) and this ranking is in agreement with Hossein-Zadeh [13] (9,186 kg vs 10,386 kg and 10,000 kg, respectively) which may be due to partition of nutrition for growth and milk production in lactation 1 cows. The highest PS in lactation 1 was in agreement with Gengler [14] who reported higher persistency in first lactation than the other lactations. In the present study, FP increased over the three lactations while PP was steady across all three lactations.

### Heritability

The heritability of MILKW was highest in lactation 1 compared to lactation 2 and 3 (0.21 vs 0.01 and 0.08, respectively) while for MILKTI, heritability is similar between lactation 1 and 3 but lower in lactation 2 (0.19, 0.12, and 0.19, respectively). The low heritability of MILKW in lactation 2 was due to a very low additive genetic variance estimate with a relatively larger standard error, which may have been due to data limitations (small sample size) rather than reflecting a true biological result, which would be consistent with the results of Hammami et al [15] who reported similar heritability estimates across the first three lactations. The heritability estimates of MILKW and MILKTI in lactation 1 is comparable to those reported by Mohammed et al [16] (0.24) and Boonkum and Duangjinda [17] (0.23). However, the heritability of MILKW and MILKTI in lactation 1 in this herd is lower than other studies (0.35: König et al [18], 0.43: Seangjun et al [19], 0.34: Sarakul et al [20]). The lower milk yield heritability in this study compared with other studies may be due to a small additive genetic variance and/or a high residual variance, suggesting this trait was highly affected by the environmental factors such as farm and feed management, hot and humid tropical environment. In addition, the different size of the data set and the models used for analysis also may have an effect on estimation of variance components and hence heritability. Nonetheless, milk yield calculated from both methods show the possibility of improving by a selection program. The heritability estimates of lactation curve traits (PMILK, DPMILK, and PSMILK) were low to moderate within the range from 0.00 to 0.23 for lactation 1 to 3. The heritability of PMILK and DPMILK in lactation 1 was similar to that reported by Chegini et al [21] (0.23 vs 0.26 and 0.10 vs 0.10) although PSMILK was higher (0.14 vs 0.05). In general, the heritability of lactation curve traits for all lactations was quite low except the moderate heritability of PMILK in lactation 1 and 3 (0.23 and 0.17, respectively) which means PMILK can be improved by selective breeding while DPMILK and PSMILK trait need an improvement of environmental management. The heritability of fat percentage in lactations 1 and 3 were lower (0.08 and 0.07) than lactation 2 (0.24). The heritability of FP in lactation 1 was lower than most other studies e.g. Welper and Freeman [22] (0.51), Boujenane [23] (0.39), Harris and Pryce [24] (0.48), Kim et al [25] (0.41) and Koonawootrittriron et al [26] (0.22). The heritability of PP in lactation 1 was higher than lactation 2 and 3 (0.45 vs 0.22 for both) and was in the range reported in earlier studies [22] (0.55), [24] (0.52) and [25] (0.43).

### Genetic and phenotypic correlations

The genetic correlation between MILKW and MILKTI was high in lactations 1 and 2 (0.98 and 0.99) but medium in lactation 3 (0.45). The genetic correlation of MILKW with PMILK, DPMILK, and PSMILK for cows in lactation 1 (0.94, 0.57, and 0.57) were comparable with genetic correlations of 305-day milk yield with peak yield, days in milk at peak yield and persistency reported by Chegini et al [21] (0.97, 0.52, and 0.44, respectively) which also used the Wood model for calculation of milk yield and lactation curve traits in Iranian Holstein cows. However, the genetic correlation of MILKTI with lactation curve traits for cows in lactation 1 was high, ranging from 0.70 to 0.86. The high correlation of MILKW and MILKTI with PMILK in lactation 1 was similar to other studies [27–29]. Therefore, the selection of high peak milk yield would tend to improve milk production in these herds although DPMILK and PSMILK would not improve much due to low genetic correlation of both traits with PMILK (0.32 and 0.33). The genetic correlation of MILKW and MILKTI with FP in lactation 1 was low and positive (0.24 and 0.23) and with PP was medium and negative (−0.40 and −0.46) while many studies reported the negative correlation between milk yield with FP and PP [22,24,30]. Selection for high milk production might slightly decrease protein percentage.

The genetic correlations of the same trait between lactations (Table 7) were high for all the traits, ranging from 0.75 to 0.99, although the correlations for DPMILK, PSMILK and FP between lactation 1 and 3, and lactation 2 and 3, could not be estimated as the models did not converge. The high genetic correlation estimates of all traits between lactations suggested that the selection of animals for first lactation curve traits in the herd will improve traits in second and third lactations as well, although the phenotypic correlations between lactations for most of the traits were low and negative for PS.

### Genetic trend

The genetic trend of sire and cow EBVs over year of birth for all traits showed inconsistent trends in these three herds. In cows there was some decline in EBVs in recent years. However, the sire EBVs showed a slight improvement in the last 10 years after 2002. The use of these bulls may have led to the genetic gain of cows after 2012. However, this will require the analysis of data beyond 2012 to assess the genetic improvement in the cows. Overall, the results on genetic trend suggested that the selection and use of breeding bulls has not been effective. This is in spite of the fact that the selection of bulls has been made based on EBVs. There could be a number of factors which might have impacted the expected genetic gains in these herds. The limitation of financial support, inconsistent animal management and feeding practices might be the issues causing the problems in these herds. In addition, the limitation of selection, semen used, an unintentional culling of high producing cow due to health problems, and an ineffective breeding plan might also impact the genetic progress in these herds. To improve the breeding program, these issues should be examined while establishing a long-term plan by the Department of Livestock Development (DLD) or Ministry of Agriculture and Cooperatives (MOAC) to improve the production status in these herds, in conjunction with the research farm management. Based on the moderate heritability estimates of milk production traits, the selective breeding is expected to result in effective genetic progress in these herds.

In Thailand, most of the data are collected from farms under the support of the DLD and the Dairy Farming Promotion Organization of Thailand and are analysed separately by these two main organizations, although some data analysis is from other sources such as private and educational farms. These organizations publish annual sire summaries and which are mostly used by the contributing farm members. However, to help speed up and improve milk production in the country, Thailand should establish a central organization for managing and analysing the data of dairy cattle across Thailand. In addition, data on a wider variety of traits should be collected and analysed in the form of selection indices which farmers can consider and choose based on their own farm needs.

## Figures and Tables

**Table 1:** Descriptive summaries of the data for all the traits from lactation 1 to 3 of Thai dairy cattle

Trait^1)^	Lactation 1	Lactation 2	Lactation 3
MILKW (kg)	3,348±1,282 (1,210; 8.9)	3,685±1,503 (906; 8.8)	3,564±1,317 (636; 8.6)
MILKTI (kg)	3,379±1,409 (1,301; 8.9)	3,682±1,604 (949; 8.8)	3,599±1,534 (665; 8.6)
PMILK (kg)	14.95±4.97 (1,210; 8.9)	17.52±6.48 (906; 8.8)	17.49±6.09 (633; 8.6)
DPMILK (d)	49±26 (1,209; 8.9)	39±18 (904; 8.8)	41±17 (638; 8.6)
PSMILK	6.68±0.48 (1,209; 8.9)	6.43±0.39 (906; 8.8)	6.43±0.41 (638; 8.6)
FP (%)	3.55±0.66 (1,141; 7.5)	3.64±0.68 (866; 7.6)	3.64±0.62 (628; 7.7)
PP (%)	3.04±0.25 (1,087; 6.9)	3.04±0.26 (812; 6.9)	3.04±0.25 (583; 6.9)

Mean±standard deviation (number of observations; average number of test days/lactation).

1) MILKW, Wood model milk yield; MILKTI, test interval milk yield; PMILK, peak milk yield; DPMILK, days to peak milk yield; PSMILK, persistency; FP, fat percentage and PP, protein percentage.

**Table 2:** Estimates of additive genetic ( $\sigma_{A}^{2}$) and residual variance ( $\sigma_{e}^{2}$) and heritability (*h*^2^) for milk traits of Thai dairy cattle for lactations 1 to 3 using univariate models

Trait^1)^	$\sigma_{A}^{2}$	$\sigma_{e}^{2}$	*h* *^2^*
Lactation 1			
MILKW (kg)	194,382±60,152	741,880±56,789	0.21±0.06
MILKTI (kg)	201,745±65,700	863,497±62,635	0.19±0.06
PMILK (kg)	2.32±0.67	7.80±0.62	0.23±0.06
DPMILK (d)	56.84±29.04	513.99±32.86	0.10±0.05
PSMILK	0.03±0.01	0.16±0.01	0.14±0.06
FP (%)	0.03±0.02	0.30±0.02	0.08±0.05
PP (%)	0.02±0.00	0.03±0.00	0.45±0.07
Lactation 2			
MILKW (kg)	9,106±54,908	1,011,868±71,855	0.01±0.05
MILKTI (kg)	146,348±92,353	1,105,479±95,236	0.12±0.07
PMILK (kg)	1.27±0.94	12.16±1.02	0.09±0.07
DPMILK (d)	3.59±13.97	257.30±18.24	0.01±0.05
PSMILK	0.01±0.01	0.13±0.01	0.04±0.06
FP (%)	0.09±0.03	0.27±0.03	0.25±0.08
PP (%)	0.01±0.00	0.04±0.00	0.22±0.08
Lactation 3			
MILKW (kg)	82,171±91,348	912,014±98,586	0.08±0.09
MILKTI (kg)	263,300±140,601	1,131,968±135,861	0.19±0.10
PMILK (kg)	2.51±1.60	12.45±1.56	0.17±0.10
DPMILK (d)	0.00±0.00	240.43±14.29	0.00±0.00
PSMILK	0.00±0.00	0.14±0.01	0.00±0.00
FP (%)	0.02±0.02	0.26±0.03	0.07±0.09
PP (%)	0.01±0.01	0.04±0.00	0.22±0.10

1) MILKW, Wood model milk yield; MILKTI, test interval milk yield; PMILK, peak milk yield; DPMILK, days to peak milk yield; PSMILK, persistency; FP, fat percentage; PP, protein percentage.

**Table 3:** Estimates of heritability and repeatability (*r*) for milk yield, lactation characteristic and milk composition of Thai dairy cattle across three lactations using repeatability models

Trait^1)^	*h*^2^±standard error	*r*±standard error
MILKW	0.14±0.04	0.30±0.03
MILKTI	0.22±0.05	0.45±0.02
PMILK	0.27±0.04	0.33±0.03
DPMILK	0.03±0.02	0.03±0.02
PSMILK	0.04±0.02	0.04±0.02
FP	0.14±0.04	0.22±0.03
PP	0.31±0.04	0.34±0.03

1) MILKW, Wood model milk yield; MILKTI, test interval milk yield; PMILK, peak milk yield; DPMILK, days to peak milk yield; PSMILK, persistency; FP, fat percentage; PP, protein percentage.

**Table 4:** Estimated genetic correlations (below diagonal) and phenotypic correlations (above diagonal) for milk traits of Thai dairy cattle from lactation 1

Trait^1)^	MILKW (1,210)	MILKTI (1,301)	PMILK (1,210)	DPMILK (1,209)	PSMILK (1,209)	FP (1,141)	PP (1,087)
MILKW	-	0.70±0.02 (1,210)	0.92±0.01 (1,210)	0.20±0.03 (1,209)	0.21±0.03 (1,209)	0.04±0.03 (1,061)	−0.11±0.04 (1,009)
MILKTI	0.98±0.08	-	0.82±0.01 (1,210)	0.23±0.03 (1,209)	0.25±0.03 (1,209)	0.11±0.03 (1,141)	−0.01±0.03 (1,087)
PMILK	0.94±0.02	0.86±0.07	-	−0.04±0.03 (1,209)	−0.07±0.03 (1,209)	0.03±0.03 (1,061)	−0.08±0.04 (1,009)
DPMILK	0.57±0.26	0.70±0.26	0.32±0.33	-	0.93±0.00 (1,209)	0.05±0.03 (1,060)	0.03±0.03 (1,008)
PSMILK	0.57±0.21	0.72±0.20	0.33±0.27	0.99±0.02	-	0.04±0.03 (1,060)	0.01±0.04 (1,008)
FP	0.24±0.29	0.23±0.32	0.14±0.30	0.29±0.47	0.17±0.41	-	0.29±0.03 (1,087)
PP	−0.40±0.14	−0.46±0.17	−0.34±0.16	−0.06±0.28	−0.21±0.22	0.34±0.23	-

Mean±standard error (number of observations).

1) MILKW, Wood model milk yield; MILKTI, test interval milk yield; PMILK, peak milk yield; DPMILK, days to peak milk yield; PSMILK, persistency; FP, fat percentage; PP, protein percentage.

**Table 5:** Estimated genetic correlation (below diagonal) and phenotypic correlation (above diagonal) for milk traits of Thai dairy cattle from lactation 2

Trait^1)^	MILKW (906)	MILKTI (949)	PMILK (906)	DPMILK (904)	PSMILK (906)	FP (866)	PP (812)
MILKW	-	0.71±0.02 (906)	0.72±0.02 (906)	0.16±0.03 (903)	0.78±0.01 (905)	0.09±0.03 (827)	0.93±0.01 (774)
MILKTI	0.99±0.33	-	0.16±0.03 (906)	−0.06±0.04 (903)	0.28±0.03 (905)	−0.01±0.03 (866)	0.02±0.04 (812)
PMILK	0.95±0.95	0.80±0.16	-	−0.04±0.03 (903)	0.28±0.03 (905)	−0.10±0.03 (827)	−0.01±0.04 (774)
DPMILK	0.96±2.65	0.21±1.18	0.92±1.92	-	0.04±0.04 (904)	−0.01±0.04 (824)	0.02±0.04 (771)
PSMILK	0.80±0.77	0.32±0.71	0.23±0.74	0.64±0.65	-	0.01±0.04 (826)	−0.03±0.04 (773)
FP	−0.41±1.04	0.08±0.32	−0.02±0.35	0.58±1.15	0.47±0.76	-	0.28±0.03 (809)
PP	−0.99±1.15	−0.20±0.34	−0.16±0.39	−0.57±1.02	−0.49±0.49	0.65±0.21	-

Mean±standard error (number of observations).

1) MILKW, Wood model milk yield; MILKTI, test interval milk yield; PMILK, peak milk yield; DPMILK, days to peak milk yield; PSMILK, persistency; FP, fat percentage; PP, protein percentage.

**Table 6:** Estimated genetic correlation (below diagonal) and phenotypic correlation (above diagonal) for milk traits of Thai dairy cattle from lactation 3

Trait^1)^	MILKW (636)	MILKTI (665)	PMILK (633)	DPMILK (638)	PSMILK (638)	FP (628)	PP (583)
MILKW	-	0.71±0.02 (634)	0.70±0.02 (627)	0.18±0.05 (630)	0.02±0.01 (630)	0.02±0.04 (600)	−0.03±0.05 (558)
MILKTI	0.45±0.33	-	0.78±0.02 (631)	0.27±0.04 (633)	0.28±0.04 (633)	0.05±0.04 (627)	0.08±0.04 (583)
PMILK	0.90±0.27	0.99±0.07	-	−0.06±0.04 (627)	−0.10± 0.04 (628)	−0.01±0.04 (603)	−0.01±0.05 (561)
DPMILK	0.46±0.23	0.68±0.35	0.09±1.12	-	0.93±0.01 (636)	−0.03±0.04 (598)	−0.06±0.05 (556)
PSMILK	−0.99±25.71	0.98±3.69	0.03±2.42	0.99±0.04	-	−0.03±0.04 (599)	−0.04±0.05 (556)
FP	0.98±1.14	0.99±0.63	0.98±0.84	−0.09±2.49	0.44±1.44	-	0.25±0.04 (579)
PP	−0.82±0.41	−0.14±0.40	0.25±0.39	−0.31±0.54	−0.71±0.59	−0.31±0.62	-

Mean±standard error (number of observations).

1) MILKW, Wood model milk yield; MILKTI, test interval milk yield; PMILK, peak milk yield; DPMILK, days to peak milk yield; PSMILK, persistency; FP, fat percentage; PP, protein percentage.

**Table 7:** Genetic correlation for milk yield, lactation characteristic and milk composition of Thai dairy cattle between lactation 1, 2 and 3

Trait^1)^	Lactation 1 vs lactation 2	Lactation 1 vs lactation 3	Lactation 2 vs lactation 3
MILKW	0.96±0.11	0.92±0.16	0.97±0.26
MILKTI	0.99±0.06	0.87±0.11	0.94±0.07
PMILK	0.99±0.06	0.75±0.12	0.94±0.11
DPMILK	0.92±1.07	NA	NA
PSMILK	0.85±0.74	NA	NA
FP	0.89±0.17	NA	NA
PP	0.96±0.09	0.98±0.14	0.88±0.15

NA, not available.

1) MILKW, Wood model milk yield; MILKTI, test interval milk yield; PMILK, peak milk yield; DPMILK, days to peak milk yield; PSMILK, persistency; FP, fat percentage; PP, protein percentage.

**Table 8:** Phenotypic correlation for milk yield, lactation characteristic and milk composition of Thai dairy cattle between lactation 1, 2, and 3

Trait^1)^	Lactation 1 vs lactation 2	Lactation 1 vs lactation 3	Lactation 2 vs lactation 3
MILKW	0.26±0.05	0.29±0.05	0.15±0.06
MILKTI	0.32±0.05	0.28±0.05	0.53±0.05
PMILK	0.40±0.04	0.26±0.05	0.32±0.06
DPMILK	0.08±0.05	NA	NA
PSMILK	0.06±0.05	NA	NA
FP	0.22±0.05	NA	NA
PP	0.36±0.05	0.31±0.06	0.30±0.06

NA, not available.

1) MILKW, Wood model milk yield; MILKTI, test interval milk yield; PMILK, peak milk yield; DPMILK, days to peak milk yield; PSMILK, persistency; FP, fat percentage; PP, protein percentage.

## SUPPLEMENTARY MATERIAL

Supplementary Figure S1. Boxplots of estimated breeding value (EBVs) by year of birth of sires for the Wood model cumulative 305-day milk yield in the first lactation.

Supplementary Figure S2. Boxplots of estimated breeding value (EBVs) by year of birth of sires for the test interval cumulative 305-day milk yield in the first lactation.

Supplementary Figure S3. Boxplots of estimated breeding value (EBVs) by year of birth of sires for peak milk yield in the first lactation

Supplementary Figure S4. Boxplots of estimated breeding value (EBVs) by year of birth of sires for days to peak milk yield in the first lactation.

Supplementary Figure S5. Boxplots of estimated breeding value (EBVs) by year of birth of sires for persistency in the first lactation.

Supplementary Figure S6. Boxplots of estimated breeding value (EBVs) by year of birth of sires for fat percentage in the first lactation.

Supplementary Figure S7. Boxplots of estimated breeding value (EBVs) by year of birth of sires for protein percentage in the first lactation.
