# Supplementary material for: Genetic parameters of milk and lactation curve traits of dairy cattle from research farms in Thailand
Source: Anim Biosci. 2022 May 2;35(10):1499–511. doi: 10.5713/ab.21.0559 (PMC9449387; doi:10.5713/ab.21.0559)
Supplement: Supplementary Figure S1. — Boxplots of estimated breeding value (EBVs) by year of birth of sires for the Wood model cumulative 305-day milk yield in the first lactation. [file ab-21-0559-suppl1.pdf]

Supplementary document

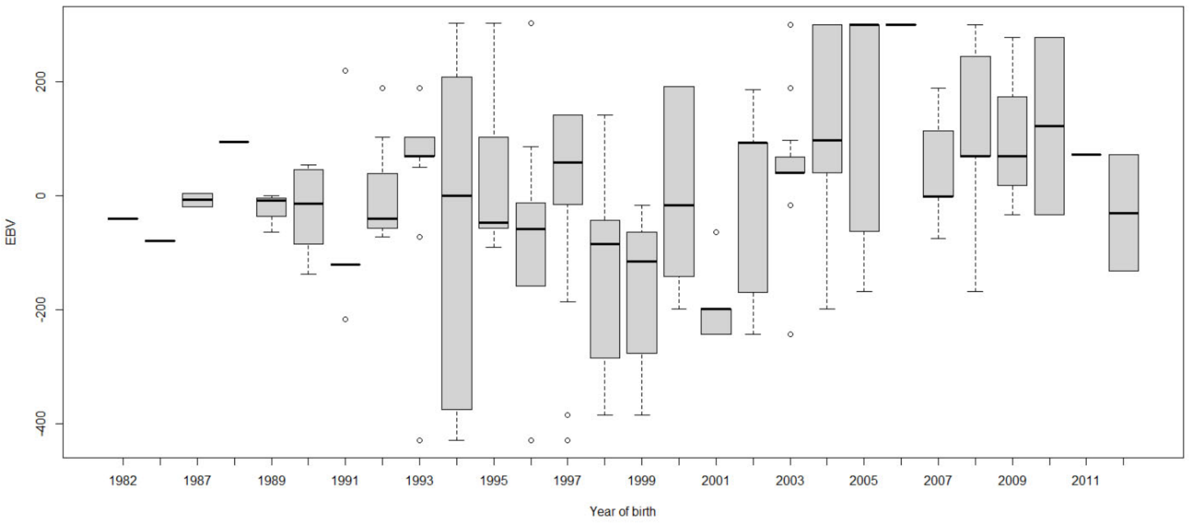

Supplementary Figure S1. Boxplots of estimated breeding value (EBVs) by year of birth of sires for the Wood model cumulative 305-day milk yield in the first lactation. The genetic trend of the Wood model cumulative 305-day milk yield EBV shows an inconsistent pattern over the year of birth. From 2002, the trend increased with fluctuation.
