# Supplementary material for: Genetic parameters of milk and lactation curve traits of dairy cattle from research farms in Thailand
Source: Anim Biosci. 2022 May 2;35(10):1499–511. doi: 10.5713/ab.21.0559 (PMC9449387; doi:10.5713/ab.21.0559)
Supplement: Supplementary Figure S4. — Boxplots of estimated breeding value (EBVs) by year of birth of sires for days to peak milk yield in the first lactation. [file ab-21-0559-suppl4.pdf]

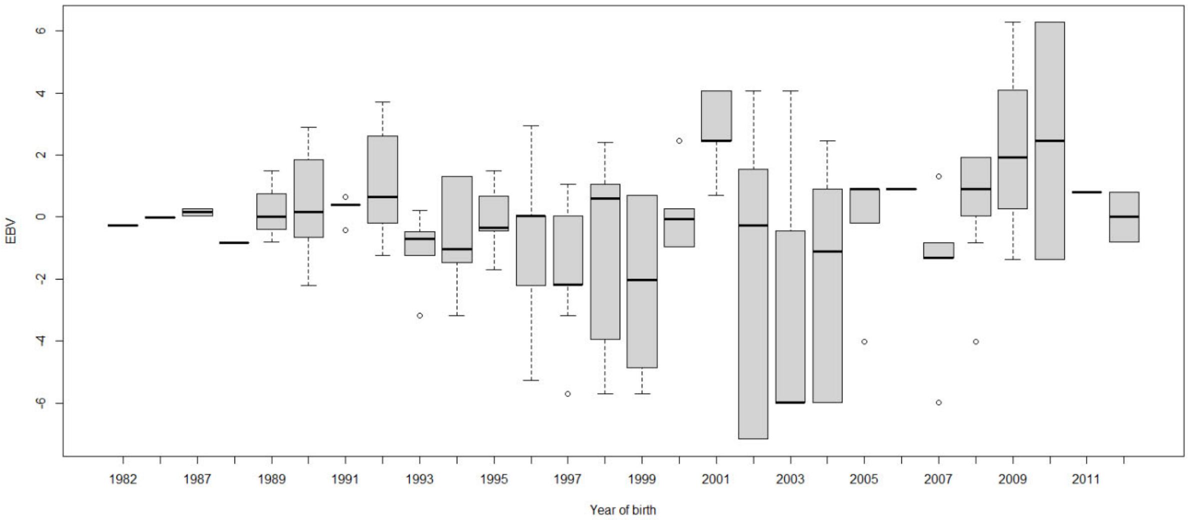

Supplementary Figure S4. Boxplots of estimated breeding value (EBVs) by year of birth of sires for days to peak milk yield in the first lactation. The genetic trend of days to peak milk yield EBV shows an inconsistent pattern over the year of birth.
