# Supplementary material for: Genetic parameters of milk and lactation curve traits of dairy cattle from research farms in Thailand
Source: Anim Biosci. 2022 May 2;35(10):1499–511. doi: 10.5713/ab.21.0559 (PMC9449387; doi:10.5713/ab.21.0559)
Supplement: Supplementary Figure S5. — Boxplots of estimated breeding value (EBVs) by year of birth of sires for persistency in the first lactation. [file ab-21-0559-suppl5.pdf]

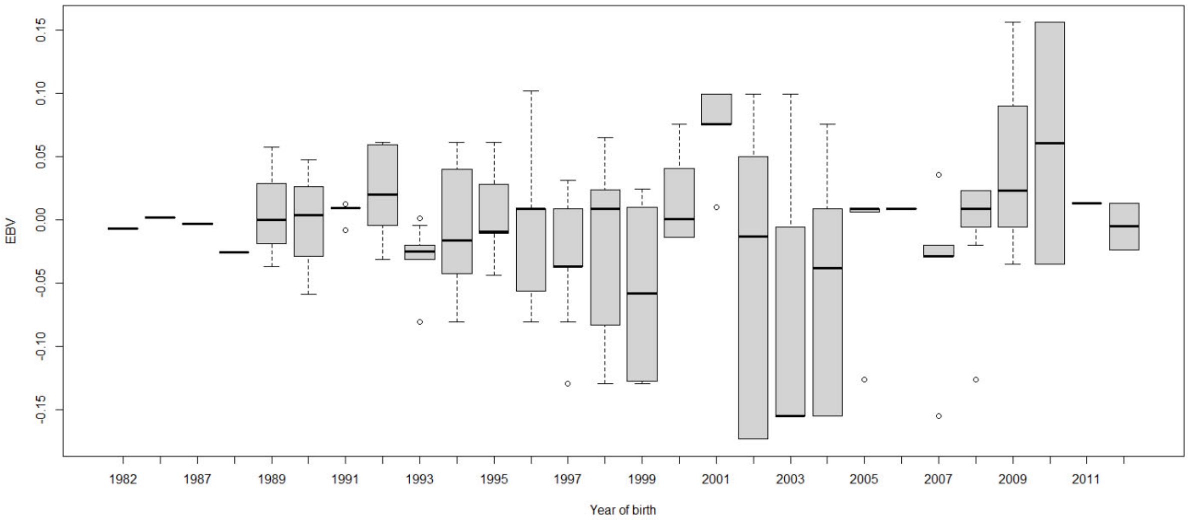

Supplementary Figure S5. Boxplots of estimated breeding value (EBVs) by year of birth of sires for persistency in the first lactation. The genetic trend of persistency EBV shows an inconsistent pattern over the year of birth.
