# Supplementary material for: Genetic parameters of milk and lactation curve traits of dairy cattle from research farms in Thailand
Source: Anim Biosci. 2022 May 2;35(10):1499–511. doi: 10.5713/ab.21.0559 (PMC9449387; doi:10.5713/ab.21.0559)
Supplement: Supplementary Figure S6. — Boxplots of estimated breeding value (EBVs) by year of birth of sires for fat percentage in the first lactation. [file ab-21-0559-suppl6.pdf]

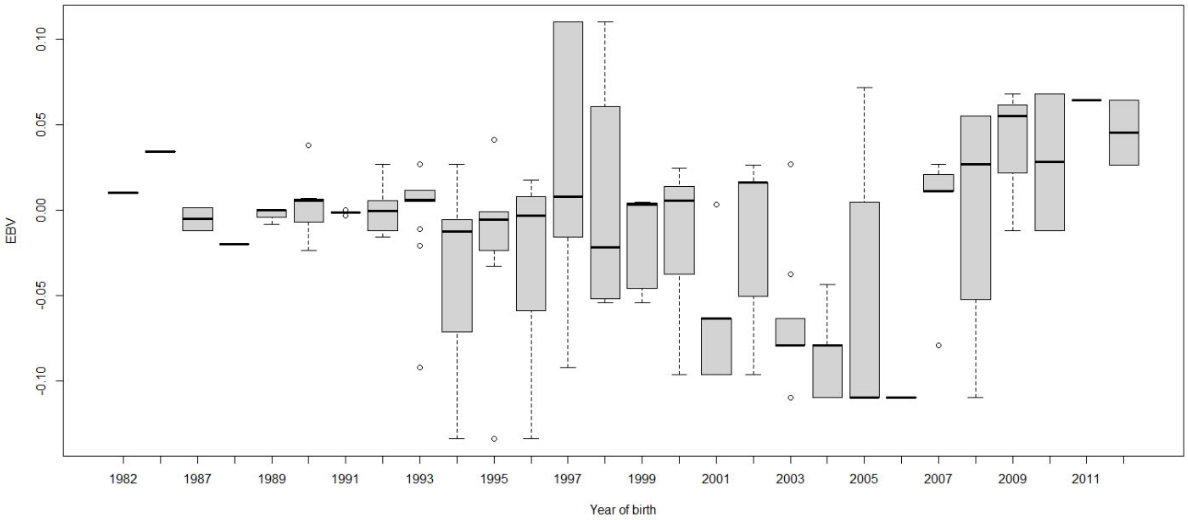

Supplementary Figure S6. Boxplots of estimated breeding value (EBVs) by year of birth of sires for fat percentage in the first lactation. The genetic trend of fat percentage EBV shows an inconsistent pattern over the year of birth. From 2007, the trend increased with fluctuation.
