# Supplementary material for: Genetic parameters of milk and lactation curve traits of dairy cattle from research farms in Thailand
Source: Anim Biosci. 2022 May 2;35(10):1499–511. doi: 10.5713/ab.21.0559 (PMC9449387; doi:10.5713/ab.21.0559)
Supplement: Supplementary Figure S7. — Boxplots of estimated breeding value (EBVs) by year of birth of sires for protein percentage in the first lactation. [file ab-21-0559-suppl7.pdf]

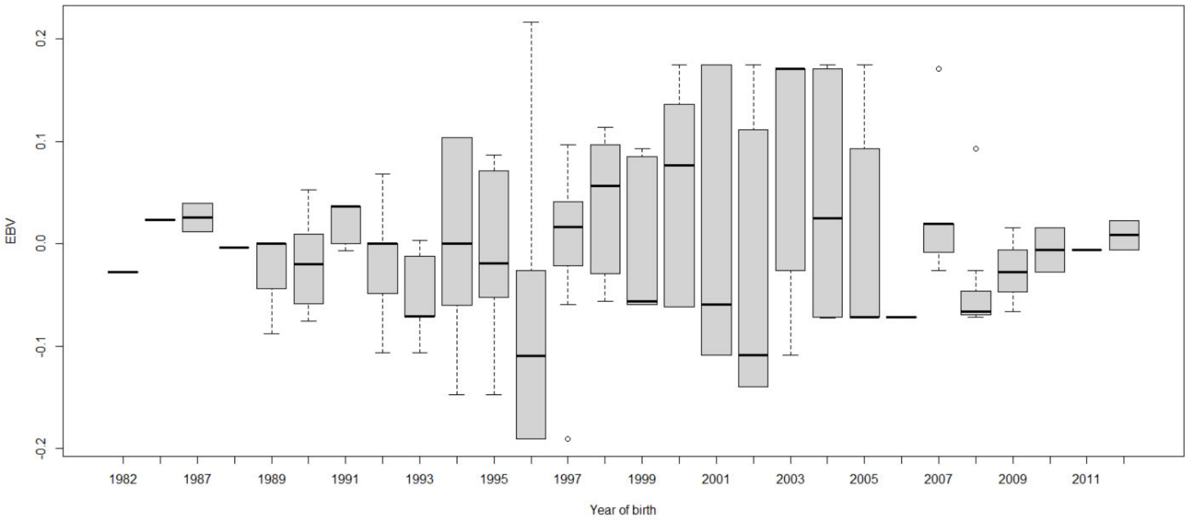

Supplementary Figure S7. Boxplots of estimated breeding value (EBVs) by year of birth of sires for protein percentage in the first lactation. The genetic trend of protein percentage EBV shows an inconsistent pattern over the year of birth.
